# Anxiety and depression among children and young people involved in family justice court proceedings: longitudinal national data linkage study

**DOI:** 10.1192/bjo.2022.6

**Published:** 2022-02-11

**Authors:** Lucy Jane Griffiths, Joanna Mcgregor, Theodora Pouliou, Rhodri D. Johnson, Karen Broadhurst, Linda Cusworth, Laura North, David V. Ford, Ann John

**Affiliations:** Population Data Science, Swansea University Medical School, UK; Population Data Science, Swansea University Medical School, UK; Population Data Science, Swansea University Medical School, UK; Population Data Science, Swansea University Medical School, UK; Centre for Child & Family Justice Research, Lancaster University, UK; Centre for Child & Family Justice Research, Lancaster University, UK; Population Data Science, Swansea University Medical School, UK; Population Data Science, Swansea University Medical School, UK; Population Data Science, Swansea University Medical School, UK

**Keywords:** Care proceedings, administrative data, data linkage, children, mental health

## Abstract

**Background:**

Little is known about mental health problems of children and young people (CYP) involved with public and private law family court proceedings, and how these CYP fare compared to those not involved in these significant disruptions to family life.

**Aims:**

This study examined records of depression/anxiety in CYP involved in public and private law proceedings using linked population-level data across Wales.

**Method:**

Retrospective e-cohort study. We calculated the incidence of primary-care-recorded depression/anxiety among CYP involved in these proceedings and in a comparison group, using Poisson regression. Depression/anxiety outcomes following proceedings were evaluated using pairwise Cox regression, with age- and gender-matched controls of CYP who had no involvement with the courts.

**Results:**

CYP in the public group had twice the risk of depression (adjusted incidence rate ratio aIRR = 2.2; 95% CI 1.9–2.6) and 20% higher risk of anxiety (aIRR = 1.2; 95% CI 1.0–1.5) relative to the comparison group. The private group had 60% higher risk of depression (aIRR = 1.6; 95% CI 1.4–1.7) and 30% higher risk of anxiety (aIRR = 1.3; 95% CI 1.2–1.4). Following private law proceedings, CYP were more likely to have depression (hazard ratio HR = 1.9; 95% CI 1.7–2.1), and anxiety (HR = 1.4; 95% CI 1.2–1.6) than the control group. Following public proceedings, CYP were more likely to have depression (HR = 2.1; 95% CI 1.7–2.5). Incidence of anxiety or depression following court proceedings was around 4%.

**Conclusions:**

Findings highlight the vulnerability of CYP involved in family court proceedings and increased risk of depression and anxiety. Schools, health professionals, social and family support workers have a role to play in identifying needs and ensuring CYP receive appropriate support before, during and after proceedings.

The Children and Family Court Advisory and Support Service (Cafcass) is a government organisation that represents children's best interests within family court proceedings, with the aim of ensuring that the welfare of the child is central in decision-making. Private law family court cases are disputes, usually between parents after relationship breakdown, about arrangements for a child's upbringing, such as where a child should live and/or with whom they should have contact. Public law family court cases are brought by local authorities and relate to the safety or welfare of children and young people (CYP). If local authorities intend to remove a child from his or her parents’ care or assume parental responsibility, they must apply for a care order. Care orders are applied for and authorised by the family courts under section 31 of the Children Act 1989. Across England and Wales, 19 037 public and 51 658 private law cases were initiated in 2018.^1^ The aim of these is to make arrangements for CYP that secure their best possible outcomes;^2^ yet, little is known about the health and well-being of those involved with family courts.

CYP involved in these proceedings may have been subject to a range of adverse experiences affecting them directly (e.g. through abuse and neglect) or indirectly, through their living environments (e.g. deprivation, exposure to parental conflict or separation, substance misuse or mental illness).^3,4^ Such events are linked to poorer short- and longer-term development and mental health,^3,5^ poorer social outcomes, educational underachievement and/or other serious disruptions to lives.^6^

There is some evidence regarding the mental health of CYP in care;^7,8^ however, to our knowledge, no studies have compared those involved in public and private law family court proceedings, and with a general population comparison group, using large-scale administrative data. This omission is concerning, given that CYP in both types of court case will have been exposed to very difficult family circumstances and disruptions. Far better evidence is needed to ensure that mental health needs are understood and taken into account in best interest decisions. Studies based on population-level data are persuasive in terms of providing policy makers and practitioners with robust evidence to shape service development.

Population-level data collected routinely by Cafcass Cymru (the Welsh Government organisation responsible for the functions of Cafcass in Wales)^9^ are available within the privacy-protecting Secure Anonymised Information Linkage (SAIL) Databank,^10^ presenting a unique opportunity for linkage to health data at the individual level to explore mental disorders in CYP involved with Cafcass Cymru. The aim of this study was to examine incidence of depression and anxiety in CYP involved in public and private family court proceedings across Wales, compared with CYP not involved in family court proceedings. Given the nature of the longitudinal data available within SAIL, a further aim was to examine risk of depression and anxiety following family court proceedings, controlling for medical histories of these conditions.

## Method

### Study design

This was a retrospective e-cohort study to investigate incidence rates (IRs) and incident rate ratios (IRRs). A matched cohort design was also used to investigate risks of depression and anxiety following initiation of court proceedings.

### Data source and linkage

All data within the SAIL Databank are treated in accordance with the Data Protection Act 2018 and are compliant with the General Data Protection Regulation. During the anonymisation of data sources within the SAIL Databank, individuals are assigned an anonymised linking field (ALF) enabling linkage of person-level data-sets.

The family justice data used for this study included the aforementioned routinely produced extract of administrative case management data maintained by Cafcass Cymru.^9^ Relevant case information for this study included: child's week of birth and gender, and the date and type of court application (public or private).

The Welsh Longitudinal General Practice (WLGP) data contain primary care records for patients registered with a Welsh general practice (GP) for approximately 80% of practices that supply data to the SAIL Databank. Each record contains information such as Read Codes (hierarchical nomenclature used by primary care physicians to record clinical summary information, i.e. medical diagnoses and symptoms) and event date (date of entry of the Read Code(s)).

Linkage was also made to the Welsh Demographic Service (WDS) data-set (an administrative register of all individuals in Wales who use the National Health Service (NHS)), for creation of our population denominator and to extract demographic information.

### Study population

**Fig. 1:**
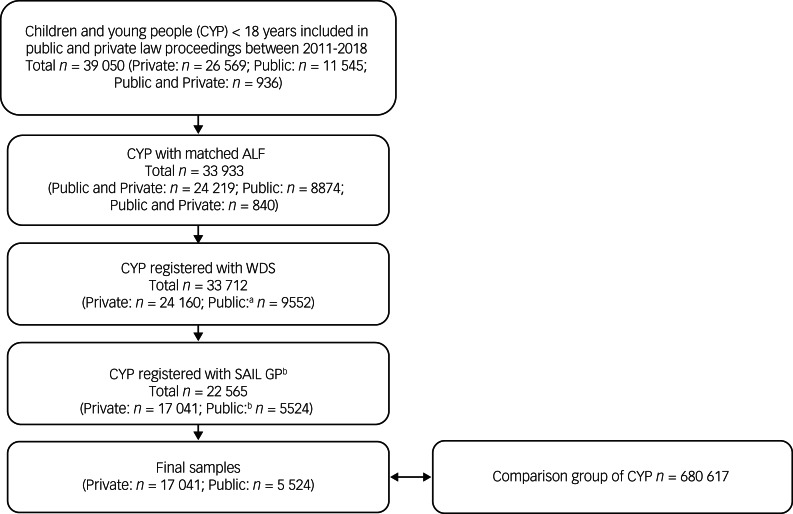
Flow diagram of study participants. a. At this stage, children and young people (CYP) involved in both public and private law proceedings were grouped with the CYP involved in public law proceedings. b. CYP who were Welsh residents, registered to a SAIL-supplying general practice (GP) between 1 January 2011 and 31 December 2018 with at least 12 months of continuous general practice data. WDS, Welsh Demographic Service.

This study included CYP involved with Cafcass Cymru between 1 January 2011 and 31 December 2018, aged <18 years at first recorded court application date. We identified 11 545 CYP involved in public law proceedings and 26 569 involved in private law proceedings. A further 936 who had been involved in both public and private law proceedings during this period were allocated to the public group. Over 86.9% (33 933 of 39 050) of these individuals were assigned an ALF enabling linkage of their information to the other data sources within SAIL. The sample was further restricted to those who had a WDS record (*n* = 33 712) and were registered with a SAIL-supplying GP for the same period with at least 12 months of continuous primary care data (*n* = 22 565). The final sample therefore consisted of 5524 CYP involved in public and 17 041 involved in private law proceedings (Fig. 1). Linkage characteristics of the Cafcass cohort in SAIL have been described elsewhere;^9^ further information on the CYP assigned an ALF (*n* = 33 933) and the final sample (22 565) are provided in supplementary Table 1 (available at https://doi.org/10.1192/bjo.2022.6), indicating greatest differences (i.e. loss of representation) in the public group for those in the two most deprived areas.

A general comparison group of all 680 617 CYP aged <18 years who were not involved in family court proceedings was selected from the SAIL Databank for the same period. From this group, we randomly selected ten controls per case matched on age and gender, resulting in a control sample of 225 650 CYP for the time-to-event analyses.

### Measures

WLGP records from 1 January 2011 to 31 December 2019 were analysed for the presence of Read Codes indicating diagnoses or symptoms of depression and anxiety based on validated code lists developed by the Adolescent Mental Health Data Platform.^11–13^ A new record of depression or anxiety was defined as an entry with no episode recorded for that condition in the previous 12 months.

Demographic information was collected from the WDS data-set. Age and residential information for each individual was collected based on the start of data collection for each year for the incidence measures and on the date of the first court application for the time-to-event analyses (as described below). Age was described according to categories of under 10 years, 10–14 and 15–17 years. The Welsh Index of Multiple Deprivation (WIMD) is the Welsh Government's official deprivation measure; WIMD 2014^14^ provides deprivation scores for small areas of Wales (lower-layer super output areas (LSOAs)), which are ranked from 1 (most deprived) to 1909 (least deprived) based on a range of domains; each LSOA contained an average population of 1600 people. These were used and grouped into quintiles for this study.

### Statistical analyses

The SAIL Databank was queried using Structured Query Language and analyses were carried out using SPSS statistical software for Windows (version 26).

#### Incidence measures

Annual incidence rates (IRs) were calculated using person-years at risk (PYAR) as a denominator. Poisson regression was used to calculate annual IRRs (incident rate ratios) and 95% confidence intervals (CIs) for the comparison, the public and the private groups and to compare the IRRs between these groups, all models adjusting for age, gender, deprivation and year. The confidence intervals were calculated using the two-tailed mid-*P* exact method, assuming Poisson distribution. The significance of the variables in the Poisson regression models was assessed using Wald tests. Robust standard errors for the estimated IRRs were used to account for clustering within general practices.

#### Time-to-event analyses

Time-to-event analyses were conducted to explore the impact of involvement in public or private court proceedings on risk of depression and anxiety. We used Cox proportional hazard regression, a method that assumes the effect on event to be constant over time, to calculate hazard ratios (HRs) with 95% confidence intervals. The HRs represent the effects of court involvement versus no court involvement on the baseline risk for either mental health condition during the follow-up period. We modelled the length of time from date of first court application (index) to the first record of depression or anxiety, or to censorship (i.e. the earliest date from: death, leaving a SAIL-registered general practice, leaving Wales or 18th birthday). We fitted separate univariate models for depression and anxiety, and multivariate models adjusting for deprivation (at index date) and previous history of these conditions as covariates. These were stratified by court application type.

### Project approvals

The project proposal was reviewed by the SAIL Information Governance Review Panel (IGRP) at Swansea University. This panel ensures that work complies with information governance principles and represents an appropriate use of data in the public interest. The IGRP includes representatives of professional and regulatory bodies, data providers and the general public. Approval for the project was granted by the IGRP under SAIL project 1040. Cafcass Cymru (the data owner of the family courts data) also approved use of the data for this project.

## Results

### Sample characteristics

Over the study period, more than three times as many CYP were involved in private than public law proceedings (*n* = 17 041 and *n* = 5524 respectively) (Table 1); 76.7% (*n* = 4236) of the public law applications were related to section 31 care proceedings. Half (51%) of public and private applications involved boys, and both public and private applications were also more common in under 10-year olds: 92.1% of the applicants in the private group fell within this age bracket, and 88.5% of the public group. Application numbers were higher for those residing in more deprived areas of Wales, almost three-fold for the private group and ten-fold for the public group in the most deprived versus the least deprived areas.

**Table 1:** Sample characteristics

	Comparison		Private court		Public court	
	*n*	%	*n*	%	*n*	%
Total	680 617		17 041		5524	
Gender						
Male	348 647	51.2	8744	51.3	2840	51.4
Female	331 970	48.8	8297	48.7	2684	48.6
Age group						
Under 10 years	461 182	67.8	15 702	92.1	4888	88.5
10–14 years	154 013	22.6	1297	7.6	600	10.9
15–17 years	65 422	9.6	42	0.2	36	0.7
Deprivation quintile^a^						
Least deprived	122 365	18.0	2142	12.6	274	5.0
Second least deprived	108 270	15.9	2311	13.6	440	8.0
Middle deprived	127 366	18.7	3018	17.7	785	14.2
Second most deprived	139 674	20.5	3984	23.4	1283	23.2
Most deprived	164 803	24.2	5272	30.9	2645	47.9

a. Missing data for deprivation (comparison group: 18 139; private: 314; public: 97).

### Incidence of depression and anxiety

#### Depression

Table 2 summarises the number of events (recorded diagnoses or symptoms), incidence rates and adjusted IRRs for depression by gender, age group, deprivation quintile and calendar year. The incidence rates for girls (private: 4.7/1000 PYAR (95% CI 4.5–4.9); public: 10.4/1000 (95% CI 9.9–11.0)) were higher than for boys (private: 2.4/1000 (95% CI 2.3–2.6); public: 2.9/1000 (95% CI 2.6–3.2)); incidence of depression was therefore also twice as high in girls, compared with boys, in private cases (IRR = 1.9 (95% CI 1.6–2.4)) and three times as high in public cases (IRR = 3.1 (95% CI 2.3–4.3)). In the comparison group, girls also had higher rates (IRR = 2.4 (95% CI 2.3–2.5)).

**Table 2:** Number of events (recorded diagnoses or symptoms) and incidence of depression among children and young people involved in private and public law proceedings, and in the comparison group

	Comparison (*n* = 680 617)			Private court (*n* = 17 041)			Public court (*n* = 5524)		
	Events, *n*	IR (95% CI)	IRR^a^ (95% CI)	Events, *n*	IR (95% CI)	IRR^a^ (95% CI)	Events, *n*	IR (95% CI)	IRR^a^ (95% CI)
Total	16 485	4.6 (4.6–4.6)		384	3.5 (3.4–3.7)		212	6.5 (6.2–6.8)	
Gender									
Male	5077	2.8 (2.7–2.8)	1	135	2.4 (2.3–2.6)	1	48	2.9 (2.6–3.2)	1
Female	11 408	6.5 (6.5–6.6)	2.4 (2.3–2.5)*	249	4.7 (4.5–4.9)	1.9 (1.6–2.4)*	164	10.4 (9.9–11.0)	3.1 (2.3–4.3)*
Age group									
Under 10 years	297	0.1 (0.1–0.2)	1	34	0.4 (0.4–0.5)	1	8	0.4 (0.3–0.5)	1
10–14 years	5322	5.2 (5.2–5.3)	36.2 (32.1–40.7)*	195	8.6 (8.2–9.0)	19.1 (13.1–27.7)*	116	14.3 (13.5–15.2)	36.3 (17.6–74.5)*
15–17 years	10 866	20.5 (20.4–20.7)	142.8 (127.1–160.5)*	155	37.3 (35.3–39.4)	75.6 (51.3–111.4)*	88	52.9 (49.3–56.9)	119.4 (57.3–248.9)*
Deprivation quintile									
Least deprived	2585	3.9 (3.8–3.9)	1	54	3.9 (3.6–4.3)	1	22	9.7 (8.4–11.3)	1
Second least deprived	2370	4.1 (4.1–4.2)	1.1 (1.0–1.2)*	44	2.9 (2.7–3.3)	0.8 (0.5–1.2)	23	7.3 (6.3–8.4)	0.8 (0.5–1.5)
Middle deprived	2886	4.3 (4.3–4.4)	1.2 (1.1–1.2)*	64	3.3 (3.0–3.6)	0.9 (0.7–1.4)	35	7.1 (6.4–8.0)	0.8 (0.5–1.4)
Second most deprived	3646	4.9 (4.9–5.0)	1.4 (1.3–1.5)*	82	3.3 (3.0–3.5)	1 (0.7–1.4)	49	6.4 (5.8–7.0)	0.8 (0.5–1.3)
Most deprived	4644	5.4 (5.4–5.5)	1.6 (1.5–1.7)*	133	4.0 (3.8–4.2)	1.3 (0.9–1.8)	79	5.7 (5.3–6.1)	0.8 (0.5–1.3)
Year									
2011	1278	2.9 (2.9–3.0)	1	8	0.7 (0.6–0.9)	1	9	2.5 (2.0–3.1)	1
2012	1747	3.9 (3.8–3.9)	1.2 (1.1–1.3)*	16	1.3 (1.1–1.5)	1.3 (0.6–3.1)	7	1.8 (1.4–2.3)	0.5 (0.2–1.4)
2013	1927	4.3 (4.2–4.3)	1.3 (1.2–1.4)*	23	1.7 (1.5–2.0)	1.5 (0.7–3.4)	18	4.4 (3.8–5.2)	1 (0.4–2.3)
2014	2004	4.4 (4.4–4.5)	1.4 (1.3–1.5)*	33	2.3 (2.1–2.6)	1.5 (0.7–3.4)	21	5.0 (4.3–5.8)	1 (0.5–2.2)
2015	2322	5.1 (5.1–5.2)	1.7 (1.5–1.8)*	57	3.9 (3.6–4.3)	2.2 (1.0–4.6)**	24	5.6 (4.9–6.4)	1 (0.4–2.1)
2016	2101	4.6 (4.6–4.7)	1.5 (1.4–1.6)*	57	3.8 (3.5–4.2)	1.8 (0.8–3.8)	41	9.5 (8.6–10.6)	1.4 (0.7–2.8)
2017	2406	5.3 (5.2–5.4)	1.8 (1.7–1.9)*	80	5.5 (5.1–5.9)	2.1 (1.0–4.4)**	38	9.1 (8.1–10.1)	1.1 (0.5–2.3)
2018	2700	6.3 (6.2–6.4)	2.1 (1.9–2.2)*	110	7.9 (7.4–8.5)	2.6 (1.3–5.4)**	54	14.0 (12.8–15.4)	1.5 (0.7–3.1)

IR, incident rate per 1000 person-years at risk; IRR, incident rate ratio; CI, confidence interval.

a. Adjusted for calendar year, gender, age and deprivation.

* *P* < 0.001; **P < 0.05 (Wald test).

Incidence of depression was also higher for older children (Table 2). Incidence was 0.4 cases per 1000 PYAR for those under 10 years of age in both private and public groups, and there was a marked age-related trend with increasing age for both the private group (e.g. at age 15–17: IR = 37.3/1000 (95% CI 35.3–39.4) and IRR = 75.6 (95% CI 51.3–111.4)) and the public group (e.g. age 15–17: IR = 52.9/1000 (95% CI 49.3–56.9) and IRR = 119.4 (95% CI 57.3–248.9)). Again, this upward trend with increasing age is reflected in the comparison group (Table 2).

Adjusted incidence rates of depression did not vary by deprivation quintile for the private or public groups (Table 2). However, for the comparison group incidence was highest in the most deprived areas (IRR = 1.6 (95% CI 1.5–1.7)) relative to the least deprived areas.

**Fig. 2:**
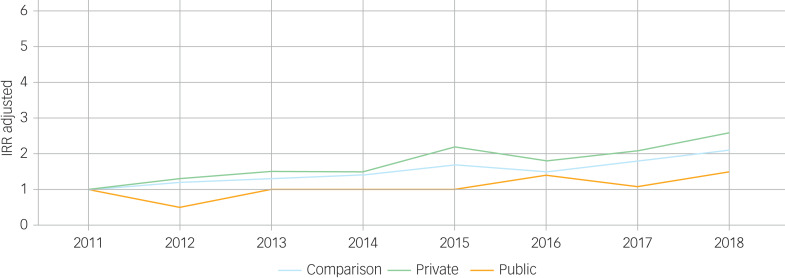
Adjusted incidence rate ratios (IRRs) of depression over time for children and young people involved in private and public law proceedings and the comparison group.

Table 2 and Fig. 2 summarise trends over time, relative to the base year 2011. For the comparison group, rates of depression increased over time, from 2.9/1000 PYAR (95% CI 2.9–3.0) in 2011 to 6.3/1000 (95% CI 6.2–6.4) in 2018. Rates of depression in the private group were almost three times as high in 2018 compared with 2011 (for 2018, IRR = 2.6 (95% CI 1.3–5.4)). For the public group, rates remained similar over time.

#### Anxiety

As for depression, compared with boys, rates of anxiety were higher in girls involved in private (IR = 5.4/1000 PYAR (95% CI 5.2–5.6); IRR = 1.6 (95% CI 1.3–1.9)) and in public (IR = 5.9/1000 (95% CI 5.5–6.3); IRR = 1.5 (95% CI 1.1–2.1)) proceedings (Table 3). Again, incidence of anxiety increased with increasing age across all groups (Table 3). The incidence of anxiety stood at around 5 cases per 1000 PYAR across all groups (private, public and comparison group), again with little difference according to deprivation quintile (Table 3).

**Table 3:** Number of events (recorded diagnoses or symptoms) and incidence of anxiety among children and young people involved in private and public law proceedings, and in the comparison group

	Comparison (*n* = 680 617)			Private court (*n* = 17 041)			Public court (*n* = 5524)		
	Events, *n*	IR (95% CI)	IRR^a^ (95% CI)	Events, *n*	IR (95% CI)	IRR^a^ (95% CI)	Events, *n*	IR (95% CI)	IRR^a^ (95% CI)
Total	17 815	5 (4.9–5)		470	4.3 (4.2–4.5)		152	4.7 (4.4–4.9)	
Gender									
Male	6238	3.4 (3.4–3.4)	1	185	3.3 (3.1–3.5)	1	59	3.5 (3.2–3.8)	1
Female	11 577	6.6 (6.6–6.7)	2 (1.9–2)*	285	5.4 (5.2–5.6)	1.6 (1.3–1.9)*	93	5.9 (5.5–6.3)	1.5 (1.1–2.1)**
Age group									
Under 10 years	2197	1.1 (1.1–1.1)	1	141	1.7 (1.6–1.8)	1	24	1.1 (0.9–1.2)	1
10–14 years	7314	7.1 (7.1–7.2)	6.7 (6.4–7)*	238	10.4 (10–10.9)	4.8 (3.9–6)*	90	11.1 (10.3–11.9)	10.2 (6.4–16.2)*
15–17 years	8304	15.7 (15.6–15.8)	14.7 (14–15.4)*	91	21.9 (20.4–23.5)	8.6 (6.5–11.4)*	38	22.8 (20.5–25.5)	18.7 (10.9–32.1)*
Deprivation quintile									
Least deprived	3445	5.2 (5.1–5.2)	1	71	5.2 (4.8–5.6)	1	14	6.2 (5.2–7.4)	1
Second least deprived	2860	5 (4.9–5.1)	1 (0.9–1)	72	4.8 (4.5–5.2)	1 (0.7–1.3)	15	4.7 (4–5.7)	0.9 (0.4–1.8)
Middle deprived	3375	5 (5–5.1)	1 (1–1.1)	89	4.6 (4.3–4.9)	0.9 (0.7–1.3)	29	5.9 (5.2–6.7)	1.1 (0.6–2)
Second most deprived	3625	4.9 (4.9–5)	1 (1–1.1)	112	4.5 (4.2–4.7)	0.9 (0.7–1.3)	28	3.6 (3.2–4.1)	0.7 (0.4–1.4)
Most deprived	4110	4.8 (4.7–4.8)	1 (1–1.1)	110	3.3 (3.1–3.5)	0.7 (0.5–1)**	62	4.5 (4.1–4.9)	0.9 (0.5–1.7)
Year									
2011	1130	2.6 (2.5–2.6)	1	9	0.8 (0.7–1)	1	6	1.7 (1.3–2.2)	1
2012	1362	3 (3–3.1)	1.1 (1–1.2)**	25	2 (1.8–2.3)	2.3 (1.1–4.9)**	14	3.6 (3–4.3)	1.8 (0.7–4.7)
2013	1622	3.6 (3.5–3.7)	1.3 (1.2–1.4)*	27	2 (1.8–2.3)	2.1 (1–4.5)	12	2.9 (2.4–3.6)	1.3 (0.5–3.4)
2014	1978	4.4 (4.3–4.4)	1.6 (1.5–1.8)*	33	2.3 (2.1–2.6)	2.1 (1–4.4)	15	3.5 (3–4.2)	1.3 (0.5–3.5)
2015	2336	5.2 (5.1–5.2)	2 (1.8–2.1)*	53	3.6 (3.3–4)	3 (1.5–6.1)**	13	3 (2.5–3.7)	0.9 (0.4–2.5)
2016	2593	5.7 (5.6–5.8)	2.2 (2.1–2.4)*	80	5.4 (5–5.8)	4 (2–8.1)*	32	7.4 (6.6–8.4)	2.1 (0.9–5.1)
2017	3181	7 (6.9–7.1)	2.7 (2.5–2.9)*	95	6.5 (6.1–7)	4.5 (2.2–8.9)*	32	7.6 (6.8–8.6)	2 (0.8–4.8)
2018	3613	8.4 (8.3–8.5)	3.2 (3–3.4)*	148	10.7 (10.1–11.3)	6 (3.1–11.9)*	28	7.3 (6.4–8.3)	1.8 (0.7–4.3)

IR, incident rate per 1000 person-years at risk; IRR, incident rate ratio; CI, confidence interval.

a. Adjusted for calendar year, gender, age and deprivation.

* *P* < 0.001; **P < 0.05 (Wald test).

**Fig. 3:**
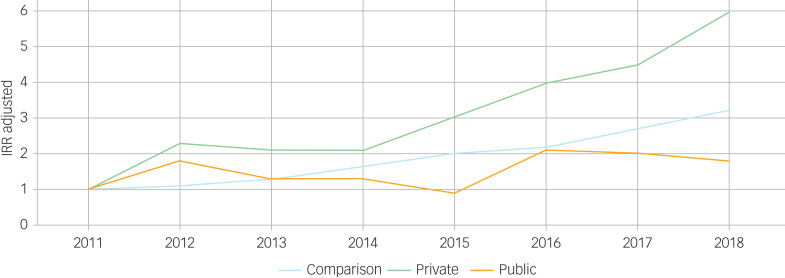
Adjusted incidence rate ratios (IRRs) of anxiety over time for children and young people involved in private and public law proceedings and the comparison group.

Trends over time for anxiety are shown in Table 3 and Fig. 3. There was a significant increase in the incidence of anxiety from 2011 to 2018 for the private group: from IR = 0.8/1000 PYAR (95% CI 0.7–1.0) cases in 2003 to IR = 10.7/1000 (95% CI 10.1–11.3) in 2018 (IRR = 6 (95% CI 3.1–11.9)). For the public group, rates of anxiety (ranging from IR = 1.7/1000 (95% CI 1.3–2.2) in 2003 to a high of IR = 7.6/1000 (95% CI 6.8–8.6) in 2017) were fairly stable over the study period (e.g. for 2018, IRR = 1.8 (95% CI 0.7–4.3)). In contrast, for the comparison group, rates of anxiety were almost three-fold in 2018 (IR = 8.4/1000 (95% CI 8.3–8.5)) compared with 2011 (IR = 2.6/1000 (95% CI 2.5–2.6)) with IRR = 3.2 (95% CI 3.0–3.4).

#### Comparing public, private and comparison group

**Fig. 4:**
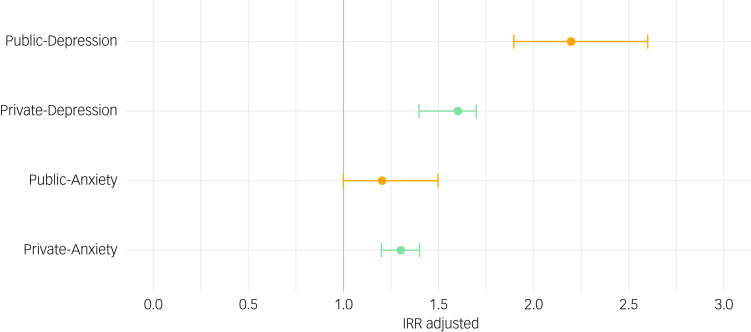
Adjusted incidence rate ratios (IRRs) of anxiety and depression for children and young people involved in private and public law proceedings.

Overall, incidence of depression was higher in the public group (IR = 6.5/1000 PYAR (95% CI 6.2–6.8)) than in the private group (IR = 3.5/1000 (95% CI 3.4–3.7)) and the comparison group (IR = 4.6/1000 (95% CI 4.6–4.6)) (Table 4 and Fig. 4). However, as can be seen from the adjusted IRRs, rates of depression were twice as high (IRR = 2.2 (95% CI 1.9–2.6)) in the public group and 60% higher in the private group (IRR = 1.6 (95% CI 1.4–1.7)), compared with the comparison group.

**Table 4:** Total number of events (recorded diagnoses or symptoms) and incidence of depression and anxiety among children and young people involved in private and public law proceedings, and the comparison group

	Depression			Anxiety		
	Events, *n*	IR (95% CI)	IRR^a^ (95% CI)	Events, *n*	IR (95% CI)	IRR^a^ (95% CI)
Total	17 081			18 437		
Comparison	16 485	4.6 (4.6–4.6)	1 (0–0)	17 815	5 (4.0–9–5)	1 (0–0)
Private court	384	3.5 (3.4–3.7)	1.6 (1.4–1.7)*	470	4.3 (4.2–4.5)	1.3 (1.2–1.4)*
Public court	212	6.5 (6.2–6.8)	2.2 (1.9–2.6)*	152	4.7 (4.4–4.9)	1.2 (1.0–1.5)*

IR, incident rate per 1000 person-years at risk; IRR, incident rate ratio; CI, confidence interval.

a. Adjusted for calendar year, gender, age and deprivation.

* *P* < 0.001 (Wald test).

Incidence of anxiety was slightly lower in the public (IR = 4.7/1000 PYAR (95% CI 4.4–4.9)) and private (IR = 4.3/1000 (95% CI 4.2–4.5)) groups than in the comparison group (IR = 5.0/1000 (95% CI 4.9–5.0)). Following adjustment, rates were 30% higher in the private group (IRR = 1.3 (95% CI 1.2–1.4)) and 20% higher in the public group (IRR = 1.2 (95% CI 1.0–1.5)).

### Time-to-event

Characteristics of the cohort and matched control group for the time-to-event analyses are provided in supplementary Tables 2 and 3. CYP involved in private law proceedings were significantly more likely to develop depression than the control group (HR = 1.9 (95% CI 1.7–2.1)) and this was also evident in boys and girls separately (Table 5). Similarly, they were more likely to have anxiety (HR = 1.4 (95% CI 1.2–1.6)). CYP involved in public law proceedings were also subsequently more likely to have depression than the control group (HR = 2.1 (95% CI 1.7–2.5)) but not anxiety (HR = 1.2 (95% CI 0.9–1.4)). Incidence proportions are also shown in Table 5: 4.2% of the private and 4.4% of the public cohorts had a new health record for anxiety or depression following court proceedings.

**Table 5:** Time-to-event analyses for anxiety and depression among children and young people (CYP) before and after private and public law proceedings

		Anxiety				Depression			
	CYP, *n*	Events, *n* (%)	History of anxiety,^a^ *n* (%)	Unadjusted HR (95% CI)	Adjusted^b^ HR (95% CI)	Events, *n* (%)	History of depression,^c^ *n* (%)	Unadjusted HR (95% CI)	Adjusted^d^ HR (95% CI)
Private court									
All	17 041	384 (2.3)	13 (3.4)	1.4 (1.2–1.5)*	1.4 (1.2–1.6)*	328 (1.9)	15 (4.6)	2.0 (1.7–2.2)*	1.9 (1.7–2.1)*
Male	8744	148 (1.7)		1.4 (1.2–1.7)*	1.4 (1.2–1.7)*	113 (1.3)		2.1 (1.7–2.5)*	2.0 (1.7–2.5)*
Female	8297	236 (2.8)		1.4 (1.2–1.6)*	1.6 (1.2–1.6)*	215 (2.6)		1.9 (1.6–2.2)*	1.8 (1.6–2.1)*
Public court									
All	5524	100 (1.8)	10 (10.0)	1.2 (1.0–1.5)	1.2 (0.9–1.4)	141 (2.6)	14 (9.9)	2.3 (1.9–2.8)*	2.1 (1.7–2.5)*
Male	2840	39 (1.4)		1.4 (1.0–1.9)	1.4 (1.0–1.9)	32 (1.1)		1.7 (1.1–2.4)**	1.5 (1.0–2.3)**
Female	2684	61 (2.3)		1.1 (0.8–1.4)	1.1 (0.8–1.4)	109 (4.1)		2.6 (2.1–3.3)*	2.3 (1.9–2.9)*

HR, hazard ratio; CI, confidence interval.

a. History of anxiety in those with a diagnosis following court proceedings.

b. Adjusted for previous history (ever) of anxiety and deprivation.

c. History of depression in those with a diagnosis following court proceedings. Numbers not provided for males and females separately owing to the small numbers (disclosure risk).

d. Adjusted for previous history (ever) of depression and deprivation.

* *P* < 0.001; **P < 0.05.

## Discussion

### Summary of main findings

The incidence of depression and anxiety recorded in primary care was higher for CYP involved in public and private family court proceedings compared with those not involved with family courts. Incidence of both recorded conditions was higher for girls and increased with increasing child age. However, adjusted rates did not vary by our measure of relative deprivation, as shown in the general population, suggesting heightened vulnerability of these CYP across the board. Regarding trends over time from 2011 to 2018, rates of depression and anxiety increased for those involved in private cases, mirroring trends in the comparison group, but they remained stable for those involved in public cases, perhaps reflecting differences in help-seeking behaviours.

The results of our time-to-event analyses, focusing on occurrence of new diagnoses or symptoms of depression or anxiety following court involvement – and taking into account previous medical history – suggests that CYP involved in private law proceedings were more likely to have depression or anxiety than the control group. Those involved in public law proceedings were subsequently more likely to have depression. Just over 4% had anxiety or depression.

### Study strengths and limitations

This is the first time that population-level family law records have been linked to health data sources in Wales to examine mental health outcomes for CYP, enabled through the SAIL Databank. However, studies based on administrative data are limited by the scope and quality of available data, which are collected primarily for administrative rather than research purposes.

Limitations of the Cafcass Cymru data-set have been previously described.^9^ We acknowledge the possibility of some selection bias, which can occur if subgroups of individuals have different linkage rates;^15^ however, 87% of the Cafcass Cymru records were successfully matched in SAIL, enabling linkage to health records, and we report on characteristics of the final sample with GP data. This study reports on problems only for CYP who had at least 12 months of GP data and who were thus included in the final sample; there is therefore a possibility that we have excluded children with poorer mental health due to residential mobility,^16^ with mobility more common in CYP involved in public law proceedings.^17^ Further, we only report on problems both known to the healthcare practitioners and coded into patient records; our figures are therefore likely an underestimate of the true numbers of CYP with anxiety and depression. The longitudinal nature of health records has, however, permitted exploration of outcomes of newly diagnosed mental health problems following court proceedings.

A study limitation is the lack of data on the study participants (e.g. ethnicity and other sociodemographic information such as parental educational level), limiting a fuller description of the cohort and our ability to adjust for these factors. Although our time-to-event analysis included deprivation data based on the date of first court application and therefore is likely to be for the family address, the incidence analysis was based on annual data and may include deprivation data based on placement address following a care order, so the latter should be interpreted with more caution in the public law population. Further, there was a lack of information regarding interventions received following involvement with the family courts. The majority of private law applications are for child arrangement orders^18^ but this study has not explored the nature of these or profiles of those involved in single or repeat cases. Similarly, we have not examined legal outcomes for those involved in public law proceedings, which may, for example, involve an order for permanent removal from parents and varying placements, such as placed for adoption. For the small proportion of participants who were adopted, NHS registration numbers will also have changed and will therefore have been lost to follow-up. The circumstances of the different orders can clearly have a wide-ranging impact on emotional health. Further analyses are therefore warranted to understand the impact of court involvement in greater depth. Acquisition of further data-sets from local authorities (such as social services) with linkage to existing data within the SAIL Databank will facilitate this future research.

### Comparison with previous literature

Recent evidence linking health and Cafcass Cymru records reports on heightened mental health problems of mothers involved in public law proceedings;^19^ no previous large-scale studies have used routine administrative data to examine or compare similar problems experienced by CYP involved in public or private family court proceedings across Wales.

Based on other study types, there is more robust evidence that parental conflict that is frequent, intense, poorly resolved and about the child is associated with multiple negative outcomes for children.^20,21^ Bream et al^22^ reported high levels of distress among children involved in parental disputes regarding child arrangements and, based on Cafcass welfare records for private family law proceedings, Macdonald^23^ reports on a lack of consideration of children's accounts in court recommendations and therefore failure in the system to identify those at risk for mental health problems.

Investigating the impact of public law proceedings on mental health, Famularo et al^24^ showed that post-traumatic stress disorder in children (aged 6–12) was correlated with other anxiety and psychotic disorders and presence of suicidal ideation. Hunt et al^25^ assessed outcomes for abused and neglected children placed in kinship care (with family or friends): more than half were manifesting emotional or behavioural difficulties. Mulcahy et al^26^ also examined change in children's adaptation and well-being after care proceedings; although this improved, resolving the impact of maltreatment remained a complex ‘work in progress’. Ford et al^7^ combined data from Meltzer and colleagues’ surveys of looked after British children (children looked after by local authorities) and of British children in private households and found higher levels of psychiatric disorder in children in local authority care and, a more recent survey of young people in care in Wales^27^ reports lower well-being than those not in care, with those in residential care having the lowest well-being scores.

Mental health problems are of growing concern and account for a large proportion of the disease burden in young people generally; findings from the 2017 population-level survey of child and adolescent mental health in England estimated that that 1 in 12 (8.1%) 5- to 19-year-olds had an emotional disorder such as anxiety or depression;^28^ rates also increased with age, and the disorders were more common in girls and among those living in households with the lowest household incomes. Our estimate of just over 4% for anxiety and depression reflects our calculation of incidence (new cases), inclusion of younger children and, of course, CYP who presented to health services for these problems rather than self-reported estimates.

### Implications

Further work is needed to capture the full range of mental health difficulties experienced. A better understanding of substance misuse and other problems (such as self-harm) will contribute to a better understanding of the scale and depth of problems, which the family courts must take into account during proceedings and in child placement beyond proceedings. Children's mental health needs are a significant factor in placement stability/instability.^29^ Further, to complete the picture, future research should examine associations in the opposite direction, i.e. the impact of having a child with mental health problems on parental conflict, separation and, for those who cannot agree on child arrangements, private law applications.

Welsh Government is committed to mental health support for CYP.^30^ Progress is being made with schools embedding health and well-being into the curriculum and adopting a whole-school approach to support pupils. Although this may capture vulnerable CYP involved with family courts, they may also benefit from improvements within health and local government (including social services). The capacity of local primary mental health support services remains a significant concern in terms of both access to crisis and out-of-hours services across Wales and, more generally, limited support/treatment options for CYP who need help but do not meet the threshold for specialist mental health or neurodevelopmental services.^30^ The Social Services and Well-being (Wales) Act 2014 requires social care and health professionals to work together to support the needs of these vulnerable CYP. Careful thought therefore needs to be given to how the system impacts on children already experiencing heightened vulnerability and in particular whether there is a way for the system to act as a gateway to appropriate support in situations where these issues are identified. Greater mental health assessment of CYP throughout their journey in the family justice system is required, as is more training and more effective sharing of information to help services and organisations work together.

Although the overall trend in the volume of private law applications has been modestly upwards over the past decade, there has been a steeper rise in public law applications, particularly care proceedings.^18,31^ This increase in family court cases means that increasing numbers of vulnerable CYP are at risk of depression and anxiety.

## Data Availability

The data used in this study are available from the Secure Anonymised Information Linkage (SAIL) Databank at Swansea University, Swansea, UK, which is part of the national e-health records research infrastructure for Wales. Those wishing to access data should follow the application process guidelines available at: www.saildatabank.com/application-process.

## Supplementary material

For supplementary material accompanying this paper visit http://doi.org/10.1192/bjo.2022.6. click here to view supplementary material
