# Supplementary material for: Anxiety and depression among children and young people involved in family justice court proceedings: longitudinal national data linkage study
Source: BJPsych Open. 2022 Feb 11;8(2):e47. doi: 10.1192/bjo.2022.6 (PMC8867894; doi:10.1192/bjo.2022.6)
Supplement: Supplementary file 1 [file S2056472422000060sup001.docx]

**Griffiths et al. Anxiety and depression among children and young people involved in family justice court proceedings: longitudinal national data linkage study.**

**SUPPLEMENTARY MATERIAL**

**S1 Characteristics of those registered with WDS (n=33,712) and registered to SAIL-supplying general practice (n=22,565) at court registration.**

|  | **Private court** | | | | **Public*** court** | | | |
| --- | --- | --- | --- | --- | --- | --- | --- | --- |
|  | **WDS*** | | **Final cohort**** | | **WDS*** | | **Final cohort**** | |
|  | **n** | **%** | **n** | **%** | **n** | **%** | **n** | **%** |
| **Total** | **24160** |  | **17041** |  | **9552** |  | **5524** |  |
| **Sex** |  |  |  |  |  |  |  |  |
| Male | 12328 | 51·0 | 8744 | 51·3 | 4953 | 51·9 | 2840 | 51·4 |
| Female | 11832 | 49·0 | 8297 | 48·7 | 4599 | 48·2 | 2684 | 48·6 |
| **Age group (at court date)** |  |  |  |  |  |  |  |  |
| under 10s | 19271 | 79·8 | 13530 | 79·4 | 7140 | 74·8 | 3838 | 69·5 |
| Ages 10-14 | 4440 | 18·4 | 3200 | 18·8 | 1915 | 20·1 | 1366 | 24·7 |
| Ages 15-17 | 449 | 1·9 | 311 | 1·8 | 497 | 5·2 | 320 | 5·8 |
| **Deprivation quintile (at court date)** |  |  |  |  |  |  |  |  |
| Least deprived | 2822 | 11·7 | 2123 | 12·5 | 632 | 6·6 | 336 | 6·1 |
| Second least deprived | 3464 | 14·3 | 2337 | 13·7 | 839 | 8·8 | 510 | 9·2 |
| Middle deprived | 4253 | 17·6 | 3042 | 17·9 | 1354 | 14·2 | 761 | 13·8 |
| Second most deprived | 5302 | 22·0 | 3883 | 22·8 | 1851 | 19·4 | 1307 | 23·7 |
| Most deprived | 6864 | 28·4 | 5169 | 30·3 | 3646 | 38·2 | 2477 | 44·8 |

* Registered with Welsh Demographic Service (WDS)

** Registered with SAIL supplying general practice with at least 1 year of continuous data within the study period.

*** Includes CYP involved in both public and private applications.

**S2 Characteristics of the cohort and matched control group for the time-to-event analyses**

|  | **Private court** | | | | **Public* court** | | | |
| --- | --- | --- | --- | --- | --- | --- | --- | --- |
|  | **Cohort** | | **Matched Controls** | | **Cohort** | | **Matched Controls** | |
|  | **n** | **%** | **n** | **%** | **n** | **%** | **n** | **%** |
| **Total** | **17041** |  | **170410** |  | **5524** |  | **55240** |  |
| **Sex** |  |  |  |  |  |  |  |  |
| Male | 8744 | 51·3 | 87440 |  | 2840 | 51·4 | 28400 |  |
| Female | 8297 | 48·7 | 82970 |  | 2684 | 48·6 | 26840 |  |
| **Age group (at court date)** |  |  |  |  |  |  |  |  |
| under 10s | 13530 | 79·4 | 135300 |  | 3838 | 69·5 | 38380 |  |
| Ages 10-14 | 3200 | 18·8 | 32000 |  | 1366 | 24·7 | 13660 |  |
| Ages 15-17 | 311 | 1·8 | 3110 |  | 320 | 5·8 | 3200 |  |
| **Deprivation quintile (at court date)** |  |  |  |  |  |  |  |  |
| Least deprived | 2123 | 12·5 | 30688 | 18·0 | 336 | 6·1 | 9904 | 17·9 |
| Second least deprived | 2337 | 13·7 | 26828 | 15·7 | 510 | 9·2 | 8765 | 15·9 |
| Middle deprived | 3042 | 17·9 | 31453 | 18·5 | 761 | 13·8 | 10373 | 18·8 |
| Second most deprived | 3883 | 22·8 | 35031 | 20·6 | 1307 | 23·7 | 11212 | 20·3 |
| Most deprived | 5169 | 30·3 | 41829 | 24·5 | 2477 | 44·8 | 13382 | 24·2 |

*Includes CYP involved in both public and private applications.

**S3 Counts, % of risk of anxiety / depression following, and previous, to court (private/public) for the gender/age matched controls.**

|  |  | **Anxiety** | | **Depression** | |
| --- | --- | --- | --- | --- | --- |
|  | **CYP (N)** | **Events**  **n (%)** | **History**  **of anxiety** ^a^  **n (%)** | **Events**  **n (%)** | **History**  **of depression** ^b^  **n (%)** |
| **Private Controls** | | | | | |
| All | 170410 | 2942 (1·7) | 103 (3·5) | 1854 (1·1) | 47 (2·5) |
| Males | 87440 | 1084 (1·2) |  | 615 (0·7) |  |
| Females | 82970 | 1858 (2·2) |  | 1239 (1·5) |  |
| **Public Controls** | | | | | |
| All | 55240 | 1015 (1·8) | 61 (6·0) | 765 (1·4) | 34 (4·4) |
| Males | 28400 | 351 (1·2) |  | 231 (0·8) |  |
| Females | 26840 | 664 (2·5) |  | 534 (2·0) |  |

^a^ History of anxiety in those with a diagnosis following court.

^b^ History of depression in those with a diagnosis following court.

Numbers not provided for males and females separately due to small numbers (disclosure risk).
